# Lessons Learned From a Case of Behcet’s Disease Presenting With Fever and Life-Threatening Venous Thromboembolism

**DOI:** 10.7759/cureus.32546

**Published:** 2022-12-15

**Authors:** Peter Conlon, Dawn Swan, Niamh O'Connell, Richard Conway

**Affiliations:** 1 Department of Infectious Diseases, St James's Hospital, Dublin, IRL; 2 Department of Haematology, St James's Hospital, Dublin, IRL; 3 Department of Rheumatology, St James's Hospital, Dublin, IRL

**Keywords:** behcet’s disease, sepsis, vte, thrombosis, fever of unknown origin

## Abstract

Infection mimics pose a challenge in the world of infectious diseases. Fever of unknown origin (FUO) requires careful consideration for a broad range of diagnoses. The answer often lies in a careful history and dedicated clinical examination. A delay in diagnosis can result in greater morbidity for the patient. We present the diagnostic challenges in a patient with an infection mimic, Behcet’s disease (BD), who presented with recurrent venous thromboembolism (VTE) and fever of unknown origin (FUO).

We present the case of a 53-year-old male of Irish Caucasian ethnicity who presented with a history of fevers and recurrent VTE at a university hospital in Dublin, Ireland. Past medical history includes schistosomiasis, which was treated following a trip to sub-Saharan Africa. Our patient was previously diagnosed with a provoked deep vein thrombosis (DVT). He went on to experience four subsequent episodes of VTE, including DVT, pulmonary embolism (PE), and cerebral venous sinus thrombosis (CVST) while on different forms of anticoagulation. On each of these occasions, there was a concern for sepsis due to fevers > 38°C and a C-reactive protein (CRP) > 200 mg/L.

The infection workup included routine laboratory tests, blood and urine cultures, CT of the abdomen and pelvis (CTAP), echocardiogram, and PET-CT, all of which were unrevealing. However, a focused clinical examination revealed evidence of subtle scrotal and oral ulceration, pustulation, and erythema at several sites in his upper limb following venesection and cannulation. In this context, a diagnosis of Behcet’s disease was considered.

A diagnosis of Behcet’s disease can only be confidently made after the exclusion of other potential etiologies. In this case, we had to consider a broad range of infectious (malaria, schistosomiasis, rickettsial disease, and endocarditis) and noninfectious (malignancy, antiphospholipid syndrome (APS), myeloproliferative disorders, and paroxysmal nocturnal hemoglobinuria (PNH)) diseases.

A delay in diagnosis comes at the cost of increased morbidity and mortality for the patient. A detailed history and clinical examination are key, in addition to a high index of suspicion.

Following the induction of high-dose steroid, our patient is doing very well on maintenance adalimumab. From an anticoagulation perspective, he is warfarinized and has not had any further episodes of VTE.

## Introduction

Autoinflammatory diseases can often be difficult to diagnose and rely upon the exclusion of other potential pathologies. A delay in diagnosis can result in greater morbidity for the patient. We present the diagnostic challenges in the case of Behcet’s disease (BD) who presented with recurrent venous thromboembolism (VTE) and fever of unknown origin (FUO).

Behcet’s disease was first documented by Hippocrates in the fifth century BC [1] and was described in the literature by the Turkish dermatologist Dr. Hulusi Behçet in the 1930s [2]. Since then, we operate off diagnostic criteria outlined by the “International Study Group for Behcet’s Disease” published in the Lancet in 1990 [2]. However, the incidence of BD in the Caucasian, Western European population is rare. The incidence of BD in the UK, who share a similar genetic heritage as Ireland, is 0.64 per 100,000. This compares to countries along the “Silk Road” with a higher incidence such as Turkey where the incidence is 421 per 100,000 [3]. As a result, a high index of suspicion is required to make a diagnosis. In addition to the low incidence of BD in the Irish population, it is a very heterogeneous disease with a wide spectrum of presentation [3]. The classical “triad” of genital ulcers, oral ulcers, and ocular disease is not always present. The BD spectrum is broad. It can include arthritis; skin manifestations such as erythema nodosum, purpura, and livedo reticularis; vasculature manifestations including venous or arterial thrombosis or aneurysms; and neurological manifestations including cranial neuropathy, sensory neuropathy, and visual disturbance. Fever is a rare feature in the presentation of BD; however, when present, it can point toward BD with vascular involvement [4].

Recurrent VTE has a broad differential including solid organ malignancy [5], myeloproliferative neoplasm (MPN) [6], antiphospholipid syndrome (APS), and paroxysmal nocturnal hemoglobinuria (PNH) [7]. However, these do not typically present with fever.

Anticoagulation is the standard of care for systemic VTE. However, VTE in BD is less well-defined [8]. The duration and choice of anticoagulant vary among countries and may be influenced by the prevalence of the disease. This case highlights the use of anticoagulation for a case of BD with severe thrombosis at presentation.

## Case presentation

We present the case of a 53-year-old Irish Caucasian male who presented, in May 2022, with a history of fevers and recurrent VTE to a university in Dublin, Ireland. Past medical history includes schistosomiasis, which was treated following a trip to sub-Saharan Africa. Our patient was previously diagnosed with a deep vein thrombosis (DVT) of the left calf and popliteal vein in March 2020. This was preceded by a long-distance flight and was treated for six months with rivaroxaban from March to August 2020. He went on to experience four subsequent episodes of unprovoked VTE, which we will describe individually (Table 1). Notably, he had no family history of VTE or miscarriage.

**Table 1:** Time of events and anticoagulation at the time of the events DVT: deep vein thrombosis, US: ultrasound, PE: pulmonary embolism, CTPA: CT pulmonary angiogram, OD: once daily, BD: twice daily

Event	Time	Anticoagulation at the time of the event	Radiology
Left calf DVT	March 2020	None	US Doppler
Left femoral DVT and PE	September 2020	None	US Doppler and CTPA
Right femoral DVT	April 2022	Rivaroxaban 10 mg OD	US Doppler
Left popliteal DVT and PE	May 2022	Rivaroxaban 20 mg OD	US Doppler and CTPA
Central venous sinus thrombosis	May 2022	Low-molecular-weight heparin 1 mg/kg BD	CT intracranial venogram

In September 2020, having discontinued anticoagulation one month prior, our patient experienced left lower limb pain and edema with associated shortness of breath. On this occasion, our patient presented to a local private hospital, and an ultrasound (US) Doppler demonstrated extension of the previously diagnosed DVT into the left femoral vein, and a CT pulmonary angiogram (CTPA) also confirmed a pulmonary embolism (PE).

Following this unprovoked second VTE, he was commenced on enoxaparin at a dose of 1 mg/kg twice daily and was referred to a hemostasis and thrombosis consultant in our hospital. He was subsequently transitioned to rivaroxaban 20 mg for six months, which was dose-reduced to 10 mg with an intended indefinite duration.

In April 2022, our patient presented to an external hospital with a painful swollen right lower limb following a short-duration flight (less than four hours) four weeks prior. A US Doppler confirmed a new right common femoral vein DVT despite reported 100% compliance with anticoagulation. Given a new DVT on 10 mg of rivaroxaban, his anticoagulation was escalated to 175 U/kg tinzaparin. Following further discussion with the hemostasis and thrombosis service, it was decided to recommence rivaroxaban at a dose of 20 mg. The option of transitioning to warfarin was discussed; however, our patient expressed a preference to remain on rivaroxaban. At this stage, the patient mentioned that he suffers from headaches and fevers with each VTE. He underwent testing for antiphospholipid syndrome while on a reduced dose of rivaroxaban. Beta-2-glycoprotein antibodies were within the normal range at 5.1 U/mL (normal range: 0-6.99 U/mL), IgG anticardiolipin antibodies were reported as low positive, and dilute Russell viper venom test (DRVVT) was high at a ratio of 1.41 (normal: 0-1.26). Anti-Xa while on low-molecular-weight heparin (LMWH) was 0.05 IU/mL. A paroxysmal nocturnal hemoglobinuria (PNH) screen was negative, and JAK2 V617F mutation was not detected.

**Figure 1:**
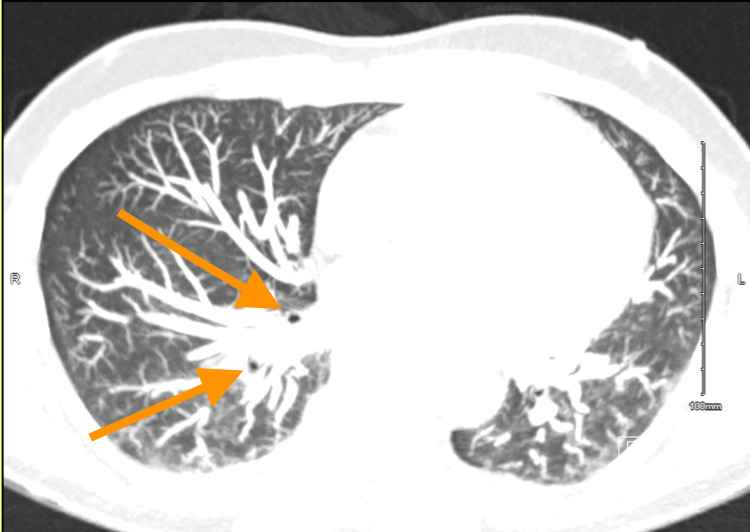
CT pulmonary angiogram demonstrating segmental and subsegmental pulmonary emboli of the right lower lobe (orange arrows) CT: computed tomography

In May 2022, the patient presented to our hospital with fevers, shortness of breath, and a swollen left lower limb. A US Doppler demonstrated a new left popliteal DVT, and a subsequent CTPA demonstrated segmental and subsegmental PEs in the right lower lobe (Figure 1).

Anticoagulation was changed to enoxaparin 1 mg/kg twice daily. In addition, there was concern over cellulitis around the left calf, and intravenous (IV) flucloxacillin was commenced. Ultimately, the plan was to transition to warfarin for indefinite anticoagulation with a target international normalized ratio (INR) of 2.5-3.5 following the completion of antibiotics.

**Figure 2:**
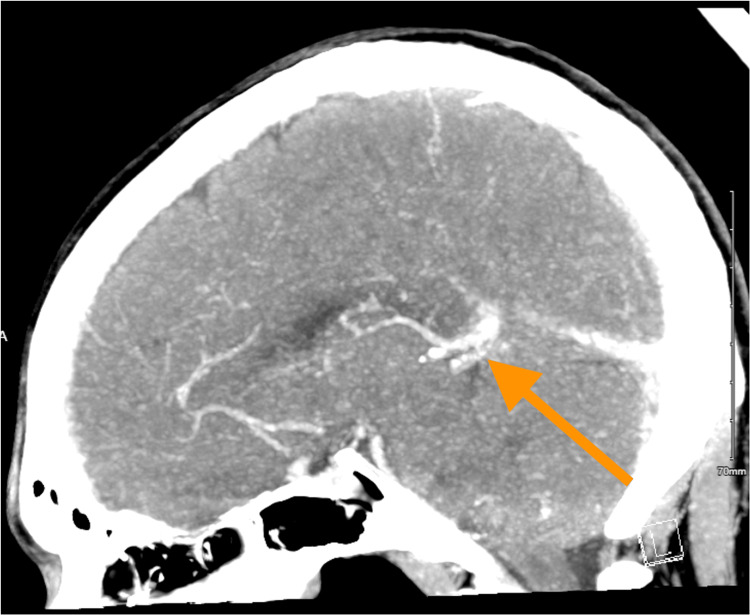
CT venogram demonstrating nonocclusive central venous sinus thrombosis (orange arrow) CT: computed tomography

Unfortunately, while taking LMWH at a dose of 1 mg/kg twice daily, he presented for a fourth time, before initiating warfarin, complaining of fever and headache. On this occasion, a CT venogram demonstrated a nonocclusive central venous sinus thrombosis (CVST) (Figure 2).

Thus far, we have a previously healthy Caucasian Irish male who has presented on several occasions with VTE at different sites including the lower limbs, lungs, and brain. In addition, he is febrile and has persistently elevated inflammatory markers. He has been investigated by the hematology service for secondary causes of VTE without a positive result. The infectious disease and rheumatology services are consulted, who drew up a broad differential, which we will discuss below.

However, on focused history, our patient revealed that over the last five years, he has been experiencing recurrent ulceration in his scrotum and oral mucosa. In addition, he begins to develop erythema and pustulation at the site of venepuncture and cannulation. This led us to consider a diagnosis of BD of the vascular phenotype.

Treatment

BD is often delayed due to diagnostic challenges in ruling out other potential pathologies. In addition to this, missing an infection can have catastrophic consequences if immunosuppression is initiated.

Following a 10-day course of IV vancomycin and ceftriaxone, corticosteroids and colchicine were initiated to manage the hyperinflammatory state. We commenced prednisolone 60 mg once daily and colchicine 500 mg twice daily. Within 48 hours, our patient’s fevers resolved, and his inflammatory markers began to normalize.

Steroids were tapered over 12 weeks, and adalimumab (an anti-tumor necrosis factor (TNF)) was introduced. The choice of an anti-TNF was based on recent data supporting anti-TNF over more traditional agents such as interferon-based regimes [9].

Outcome and follow-up

Following discharge, our patient was weaned off corticosteroids and transitioned to adalimumab. He is anticoagulated with warfarin with a target INR of 2.5-3.5.

He has not had any further episodes of VTE or fevers and is being followed closely in the rheumatology and hematology outpatient department.

## Discussion

There are some important considerations from this case. The first consideration is that we need to consider the differential for recurrent thrombotic episodes, especially while on anticoagulation, and how it changes in the context of presenting with a fever. The most common causes of recurrent VTE include malignancy [5], myeloproliferative neoplasms (MPN) [6], antiphospholipid syndrome (APS), and PNH [7].

Other potential causes include antithrombin (AT) deficiency leading to heparin resistance, vasculitides, noncompliance, inappropriate drug choice, and poor absorption.

Considering the choice and doses of anticoagulation received by our patient, compliance was reported at 100% at all times, there was no concern regarding absorption, nor was he receiving any medication that might alter drug metabolism to a clinically significant degree. Only one event occurred while receiving LMWH, and at that time, the patient had a normal AT III level of 1.01 IU/mL (reference range: 0.82-1.18 IU/mL) with an adequate peak anti-Xa (0.41 IU/mL). The activated partial thromboplastin time (aPTT) was slightly prolonged at 37.1 (reference range: 25-36.5) (Table 2). The patient underwent extensive radiological investigation including a PET-CT, which failed to identify any features suggestive of malignancy. A PNH screen was negative, and JAK2 V617F mutation was not detected. This, in combination with a normal hematocrit and essentially normal platelet count, meant that a diagnosis of an MPN was felt unlikely.

**Table 2:** Results of assays related to workup in procoagulant state aPTT: activated partial thromboplastin time, dsDNA ELISA: double-stranded DNA enzyme-linked immunoassay, PNH: paroxysmal nocturnal hemoglobinuria, LMWH: low-molecular-weight heparin

Assay	Result
aPTT (aPTT ratio)	37.1 (25-36.5) (1.3)
Beta-2-glycoprotein antibodies	5.1 U/mL (0-6.99 U/mL)
IgG anticardiolipin antibodies	Low positive (exact result not available)
dsDNA ELISA	44 IU/mL (0-9.99 IU/mL)
DNA Crithidia assay (confirmatory test)	Negative
Dilute Russell viper venom test (DRVVT)	1.41 (0-1.26)
PNH screen	Negative
JAK2 V617F mutation	Not detected
Antithrombin III level	1.01 IU/mL (0.82-1.18 IU/mL)
Peak anti-Xa level (while on LMWH)	0.41 IU/mL (0.5-1 IU/mL)

Careful consideration was required when deciding on the appropriate anticoagulation regime. We note that our patient initially had recurrent VTE on rivaroxaban and LMWH. Our routine reference range for monitoring of patients receiving 12 hourly LWMH at 1 mg/kg is 0.5-1.0 IU/mL. We think that it is important to highlight, however, that the use of anti-Xa to monitor LMWH has well-known issues [10]. Firstly, there is significant variability in results with different lots of enoxaparin [11]. Secondly, the clinical relevance of these ranges is not well proven, and additionally, different LMWHs accumulate at different rates with differing pharmacokinetics. Our patient had a peak reading of 0.41 IU/mL. This falls below our standard reference range; however, given the clinical history, demonstration of recurrent thrombosis despite at least an above-prophylactic level, and limitations of the test itself, we felt that this provided sufficient evidence of heparin failure and the requirement for an alternative anticoagulant strategy.

As mentioned, our patient did have a single positive lupus anticoagulant (LA) reading with a DRVVT of 1.46 (upper limit of normal (ULN): 1.26). This was taken while receiving LMWH (enoxaparin). While this usually only causes false-positive readings at supra-therapeutic levels, a false positive is still possible in this instance [12]. Patients with BD have also been seen to have positive LA readings, making this a nonspecific test in this case. More importantly, the patient did not have strong positive results for anticardiolipin and beta-2-glycoprotein antibodies. Recurrent thrombosis in patients on anticoagulation is common in APS, however, mostly confined to those with triple-positive disease (positivity to both antibodies and a persistently positive LA result also) [13]. To diagnose APS, a repeat DRVVT is required 12 weeks after the initial result; however, the patient has not been in a position to stop anticoagulation to facilitate this. Given the aggressively pro-thrombotic phenotype displayed by this patient, alongside features consistent with BD, but not APS, we felt, on balance, a diagnosis of BD was most plausible.

While we investigated a possible hematologic cause for recurrent VTE, we worked with our ID and rheumatology colleagues to further investigate a possible cause. From the perspective of potential infective etiologies, we had a broad differential. Given our patient’s extensive travel history, both tropical and nontropical causes were included. Routine septic screens including chest X-ray, urine analysis, and blood cultures did not reveal an obvious source.

**Figure 3:**
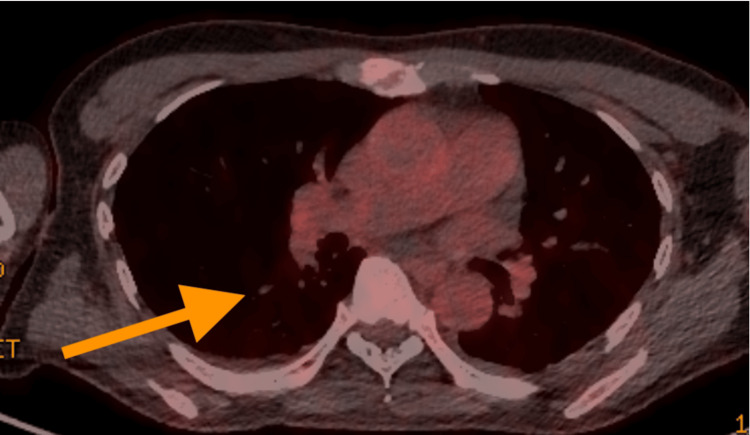
Axial PET scan of the thorax and heart demonstrating known pulmonary emboli (arrow) and no evidence of infective endocarditis PET: positron emission tomography

**Figure 4:**
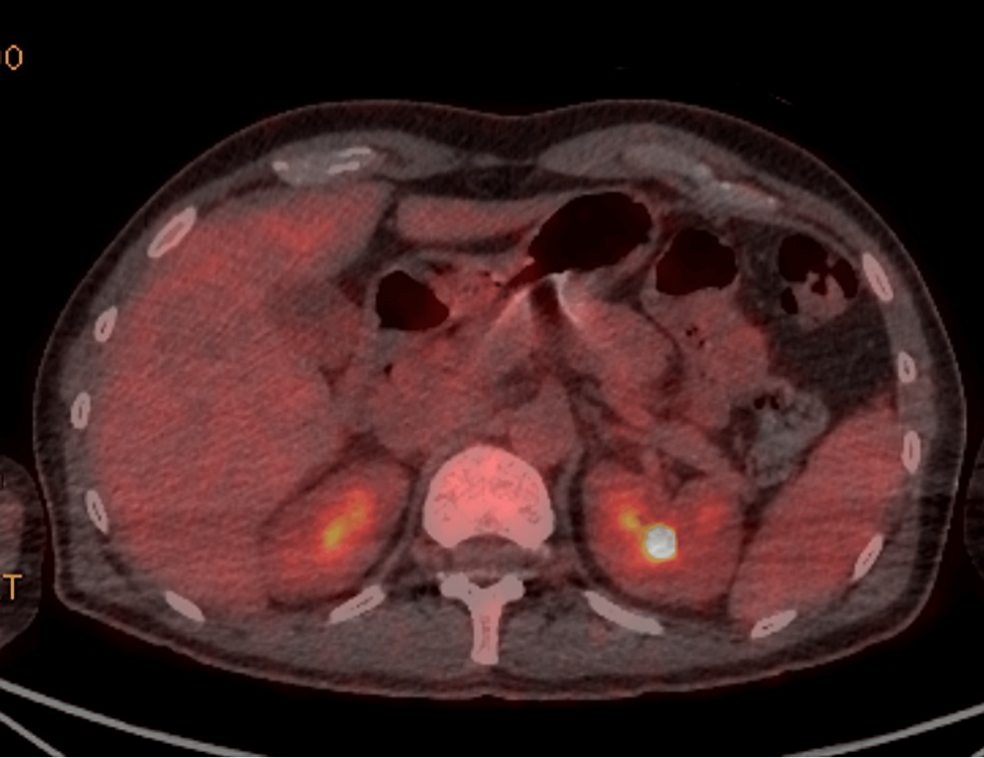
Axial PET scan through the abdomen demonstrating physiological uptake with some concern for renal infarction or infection PET: positron emission tomography

Tropical investigations took some time to get results but ultimately were unrevealing. These tests included malaria (ovale or vivax), schistosomiasis, and rickettsial disease (Table 3). In addition to this, the CT of the abdomen and pelvis (CTAP) identified potential abscess on the spleen and kidney, which raised concern over infective endocarditis (IE). However, a PET-CT did not identify any evidence of septic emboli in these organs, and it also excluded IE based on the absence of fluorodeoxyglucose (FDG) avidity in the heart (Figure 3 and Figure 4). The PET-CT also excluded any solid organ malignancy.

**Table 3:** Results of infection workup HIV Ag/Ab: human immunodeficiency virus antigen/antibody, HepB sAb: hepatitis B surface antibody, HepC Ab: hepatitis C antibody, ELISA: enzyme-linked immunoassay

Assay	Result
Blood cultures	No growth
QuantiFERON assay	Negative
HIV Ag/Ab	Negative
HepB sAb	Negative
HepC Ab	Negative
Schistosomiasis (ELISA)	Negative
Malaria rapid diagnostic test	Negative
Rickettsia serology	Negative
Syphilis serology	Negative

At this point, our suspicion was firm that a malignant or infective etiology was not driving this process. Our rheumatology colleagues suggested an autoinflammatory process. This fits with the recurrent fevers, elevated C-reactive protein (CRP), erythrocyte sedimentation rate (ESR), and ferritin, despite being clinically and hemodynamically stable throughout (Table 4).

**Table 4:** Other relevant laboratory results ESR: erythrocyte sedimentation rate, LDH: lactate dehydrogenase, CRP: C-reactive protein

Assay	Result
ESR	101 mm/hour (0-15)
Ferritin	521 ug/L (23-393)
LDH	143 IU/L (135-250)
CRP	296 mg/L (0-5)

On focused direct history, our patient revealed that over the preceding decades, he had been experiencing recurrent oral and scrotal ulceration. In addition to this, there was evidence of pustulation and erythema at several sites in his upper limb following venesection and cannulation, which represented a positive pathergy test. Combined with the extensive evidence of VTE, these findings meet the International Study Group (ISG) diagnostic criteria for BD [2].

Behcet’s disease is a rare disease among the Caucasian population in Ireland [3]. It requires a high index of suspicion and astute clinical skills to make the diagnosis. Ultimately, the diagnosis, in this case, comes after ruling out other infectious and inflammatory causes. This case had added complexity in having to consider atypical tropical diseases. Only after ruling these out can an effective management strategy be implemented. Thankfully, following the initiation of prednisolone, adalimumab, and warfarin, our patient has not experienced any further morbidity.

Within the differential for the vascular phenotype of BD is Hughes-Stovin syndrome (HSS). Both present with recurrent thrombosis, however, the characteristic features of HSS, pulmonary artery aneurysms, are not present in our case.

The second consideration is the decision to continue anticoagulation. This was made in conjunction with the rheumatology and hematology services. There is a lack of evidence to support this decision, and expert opinion is divided [9]. However, on balance, due to the severity of thrombosis at presentation at multiple sites, it was decided to continue warfarin lifelong with a target INR of 2.5-3.5. However, as the evidence evolves, this is kept under review.

## Conclusions

This case highlights the challenges faced in making a diagnosis of BD, especially in the low-incidence setting. Recurrent VTE, accompanied by fever, may point toward an autoinflammatory cause, in this case, BD. However, ultimately, a diagnosis comes after ruling out defects of the coagulation system, malignancy, and infection.

Finally, the evidence for continuing anticoagulation in BD is sparse, but in the case of severe multifocal thrombosis, in an otherwise healthy individual, it may be appropriate.
